# Application of Electricity in Medical, Surgical and Obstetric Practice

**Published:** 1859-06

**Authors:** Theophilus Mack

**Affiliations:** Lecturer on Materia Medica and Therapeutics in the Medical Department of the University of Buffalo


ART. II.—*Application of Electricity in Medical, Surgical, and Obstetric Practice.* By THEOPHILUS MACK, M. D., Lecturer on Materia Medica and Therapeutics, in the Medical Department of the University of Buffalo.

Every short period, the peaceful villages and small towns of this "happy land" (fast verging into a paradise for quacks) are wrought into a state of *excitation,* by the advent of a thaumaturgist in the healing art.

At one time, inspired by the stone which English blockheads and German visionaries have erected at Leipzig, a monument to their own credulity, "*similia similibus*," they educe the rural mind by the facility wherewith they demolish incurable disease, or evince to gaping neophites in pseudo-science, the horrors of what they are pleased to stigmatize as "the old school."

At another time a peripatetic, crowned with a patent ventilating hat, wearing electro-negative boots, electro-positive neck-tie, and endued with non-electric shirt and drawers, shows himself a very Jove in the matter of "greased lightning," and conquers all before him with the thunderbolts of his eloquence, until, all convinced

> "There is none like him in this wide world
> To speak of physic and of surgery,"

yield to the conviction, that sick maidens are exclusively medicable by sparks, nervous matrons by shocks, and the tide of human malady can only be stayed by currents. Mercury and corrosive mineral poisons are extracted in the bath, by this mysterious agent, from the victims of allopathy, almost equally as adroitly as St. John Long used to abstract the first-mentioned metal. Has it not been testified on oath, by no less a personage than a LORD, that "he saw St. John Long draw *several pounds* of a liquid, like mercury, from a patient's brain?"

Most commonly, charlatans choose some well-known remedy or method of treatment, the discovery and employment of which they arrogate to themselves. This is the case with electricity: long used and well known to the profession as a means of some power in the cure and alleviation of disease, its application is generally made by experts, in the most ignorant and often disastrous manner.

In accordance with my caption, I will submit the appliances of electricity in the medical art at the present state of science. In private practice it is found difficult to make use of but one remedy, with a view to testing its utility. The physician is generally obliged to resort to several measures contemporaneously, and often finds himself puzzled to know exactly to which to attribute his success. On this account it is extremely desirable that electricity should be introduced, and well experimented, in our hospitals, and the results carefully noted; such trials of this agent should be made, *when, ordinary therapeutic means having failed*, it should be judged opportune, and then it should be employed to the exclusion of every other medication. By a vigorous adherence to this method, a few years might enable us to speak out plainly and confidently as to the indications for electro-medication.

The administration of this remedy should always be under the immediate supervision of the medical man, because the greatest importance is to be attached to the direction, force and duration of the current—that is, for the patient's sake. Again, the fewer auxiliaries we recognize, the better; for it is an indubitable verity that every one of those assistants will soon grow self-confident, and prescribe the remedy, and administer it on his own account, and enter the arena as a competitor; and as the phlebotomist barbers of Spain, from having been entrusted to perform the numerous bleedings ordered by our brethren in that country, have assumed the right to bleed and make other prescriptions, *de jure suo*, in fact, to shave the pater familias, and do the bleeding and physicing of the whole family at a certain stipend; just so the medical electrician soon erects himself into a professional specialist, and enters upon a career of astounding cures. In this way many beneficial measures in the treatment of disease are brought into unmerited contempt at the hand of both the profession and the public; the latter agreeing with the *Diable Boiteux*, who says, "I know very well there are such things as good remedies, but cannot say whether there are any good physicians."

Every practitioner should bear in mind, that this stimulating agent is endowed with power even to a formidable degree; giving origin, sometimes, to serious nervous disturbances, tetanic spasms, and fatal cephalic congestions. If untimely used, cerebral hæmorrhage may be renewed, neuralgic and rheumatic affections recalled, when about to leave, convulsions produced, and numerous chronic diseases greatly aggravated. We are then justified in advocating much prudence and forethought in its employment generally, and especially in nervous and excitable constitutions. Caution in diagnosis is also requisite to determine the existence of lesions, which might be again aroused, or of certain chronic disorders which contra-indicate its use.

During the experiments of Van Warnen, the influence of the electric bath, or passing a current of electricity of friction from the prime conductor of an electrical machine through a person placed upon an insulated stool, was shown to be null and void; although the electric shock, or current, was clearly proved to have a very powerful effect upon living animals when passed through their bodies. In 1786, the celebrated discovery of Galvanus opened a new epoch in physics and physiology, and established rigorously the following conclusions:

1st. The passage of the electric current through the nerve, varies its excitability according to its direction. If the positive pole be placed upon a nerve, near its exit from the cranium or spinal cord, and the negative near that portion which terminates in the muscles, the current in this case is termed *direct*, the excitability of the nerve will be weakened, and even abolished; if, on the other hand, the current is made to pass through the nerve in an opposite direction, called *inverse*, the excitability of the nerve is preserved and increased.

2d. These variations in the excitability of a nerve produced by the passage of the current, tend to destroy themselves, sooner or later, on the cessation of the current; if the nerve be endowed with great irritability, as upon the living or recently killed animal, the variations cease very rapidly at the cessation of the current, while they persist for a certain time, if the nerve has already lost a great part of its excitability.

3d. The contraction excited by one current at the moment it commences to pass along the nerve differs in strength according to the direction of the current.

In a nerve, without derangement of its functions, the contraction produced by the direct current is always greater than that from the inverse current.

A battery of seven elements of Daniel's cups, is sufficient for electro-therapeutic use. In order to obtain conveniently an interrupted current, (the best in lesions of motility) at the present day, we employ only a single battery of Grove, Smee or Daniel, and introduce into the circuit an electro-magnetic coil, with a bundle of soft wires to regulate the intensity. The current is regularly interrupted by a small movable keeper of soft iron, which, upon the passage of the current, is attracted to an electro-magnet, whereby the circuit is broken; being no longer attracted, the keeper springs up and again closes the circuit. In this way a vibration of electric shocks, up to 300 per minute, can be administered.

The surface of the body to which the electrodes are applied should be moistened with a saline solution, without which precaution the patient will experience acute pain in the points touched, and the skin may be reddened, and even ulcerated.

Paralysis stands at the head of the category of maladies susceptible of improvement from the action of electricity.

Paralysis resulting from the pressure of tumors upon the trunk of a nerve, the encephalon, or spinal cord, must only be aggravated by electricity, previous to the removal of the exciting cause; but after the cure or ablation of such tumors, electricity comes to our aid, if the power of motion, &c., be not restored. In paralysis resulting from pressure of extravasated blood on the great nervous centres, the most ordinary cause of paralysis, electricity during the first months subsequent to the accident has been productive of the most disastrous results. It requires six or eight months to cicatrize the spot occupied by the clot, and that time, or even a year in bad cases, should elapse before the recovery from the injury will be sufficiently perfect, to justify the resort to electricity. The condition proper for its use will be indicated by a complete restoration of the intelligence and faculty of speech, the absence of numbness, of tingling, and particularly of painful contractions. When the proper time has arrived, if the paralyzed muscles do not contract under the influence of an energetic interrupted current, it is useless to persevere. If they contract, success is possible; but the remedy must be administered gradually, and reflex currents most carefully avoided. Each muscle should be electrized separately, for about one or two minutes, and the first operation should not last longer than about five minutes; this period can be prolonged subsequently, but ought never to exceed fifteen minutes.

The imminent danger in such cases, is the renewal of cerebral hæmorrhage, or the production of inflammation, in or about the site of former extravasation. Consequently, at the least appearance of general phenomena of contraction, the application should cease. In cases following *ramolissement*, such accidents are prone to occur, and the peril of inflammation is so great that the employment of electricity should be interdicted.

In paralysis from lesions of the medulla spinalis, the same general rules will apply. Recourse should not be had to electricity until all symptoms of inflammation are thoroughly subdued, and only if electro-muscular contractility subsists.

In paralysis from venereal excesses or rheumatism, we should try weak reflex currents, by the medium of electric pediluvia, interrupted promptly upon contraction, as such a proceeding is fraught with danger, especially in chronic myelitis.

In paralysis from injury to nervous trunks or branches, if complete loss of voluntary power and electric excitement exist, our trial must be postponed until the recovery from the lesion is perfect; then it is not very rare to see, under the influence of the current, the cicatrix become permeable and the functions reëstablished. If the paralysis be incomplete, that is to say, if voluntary motion being lost, electro-muscular contractility, or at least ordinary sensibility remain, it is a proof that the nerve or the portion of nerve distributed to the inactive muscles, has not been disorganized. In this event, as soon as we are satisfied that the current will not over-excite the sensibility, we should resort early to electricity, for the purpose of preventing atrophy of the disabled muscles.

In certain cases of atrophic paralysis, this mode of treatment in short periods, the poles applied near each other, and the currents very energetic, may prove beneficial to the local lesion.

Other varieties of paralysis capable of improvement by electricity are, the forms attending hysteria and chlorosis or severe fevers, the paralysis dependant upon saturnine and other species of poisoning, or consecutive to rheumatism. On the other hand, certain rheumatic contractions of muscles or sets of muscles are constantly treated with success by electricity, more especially by the *electric pencil.*

In all these cases, the current should be localized, and applied directly by moist conductors, or indirectly upon the nerves, so as to stimulate the paralyzed muscles and the nerves supplying them.

Chorea has been relieved occasionally, by reflex currents through the feet and hands, or upon those members of one side at a time.

The rules essential to the management of abnormal deviations in the apparatus of motility by electricity may be thus summed up:

1st. To ascertain to a certainty that there no longer exists, or did not exist, any disease in the great nervous centres.

2d. To this end we ought attentively to examine all the symptoms and carefully explore the susceptibility of the muscles to electric excitement, (muscles which preserve this quality resume voluntary motion immediately in the event of success,) by only employing weak currents at first, transmitted by moistened conductors; to suspend treatment if the sensibility is aggravated, and to prosecute it vigorously if it is only enfeebled, and to interrupt it if it becomes too much increased.

(When electro-muscular contractility is lost, it is not until atrophy has been removed and nutrition reëstablished, that voluntary motion is restored.)

3d. To give preference, when possible, to localized electricity, and to avoid, unless there be indications to the contrary, of a positive nature—*reflex currents.*

In anæsthesia, artificially produced, patients about to succumb have been preserved by interrupted currents through the phrenic nerves, or by the mouth and anus. Cutaneous anæsthesia should be treated by dry frictions with the electric hand, or by the pencil passed over the surface. Muscular anæsthesia should be combated by moistened poles, both passed over the affected muscles; the currents should be strong, and the breaking of contact frequent. Amaurosis and deafness are the lesions of sensibility in the organs of the senses to which electricity has been most frequently applied.

In paralysis of the retina or first branch of the fifth pair, electro-puncture has been resorted to, or moist conductors are placed one (the positive) upon the upper eyelid; the other, passed over the ramifications of the fifth pair. The currents should be given without the induction coil, very gently.

In deafness, enough water is poured into the meatus to fill it one-half, and the head is inclined to the opposite side, one of the wires is brought into contact with the liquid, the other is applied wet to the nuchæ. A strong current may be used.

Unbroken currents have proved successful in hyperæsthesia following rheumatism or hysteria. Electric whipping or rapid touches with energetic currents, upon the anterior and superior part of the thorax, in angina pectoris, asthma, intercostal and diaphragmatic rheumatism, has been highly praised.

Continuous currents in neuralgia, applied by wet sponges, the negative pole to the root, the positive to the painful branches of the nerves, have hitherto succeeded best. By exhausting the irritability of over-excited nerves, electricity has given favorable results, continued currents are directed by the positive pole, with or without needles, to the ramifications, and the negative pole is placed over, or in contact with the root of the nerve.

Electro-puncture, in alterations of nutrition, goitre, enlarged glands, &c., has most success by passing continued currents through numerous needles introduced into the substance of the tumor, and placing the electrodes in contact with them alternately.*

There is reason to hope beneficial results from the use of the continued current in tetanus.

Magneto-electricity has been successfully resorted to, to promote absorption of effused fluid, as in *hydrocele.*

Galvanism in aneurism at the bend of the elbow, has been very successful.

The most approved mode of applying electricity in the cure of aneurism is to introduce a needle into the centre of the tumor from its upper extremity, and to apply at its inferior margin a plate of metal, with a flannel disc soaked in an acidulated fluid beneath it. The needle should be in connection with the positive pole of the pile, and the metallic plate with the negative pole; the *séance* should last about fifteen minutes.

Before the operation, the circulation should be stopped by compression, above and below the tumor.

The eschars apt to follow in the points where the needles penetrate the skin, may be obviated by coating them (the needles) with shell-lac up to a short distance from the point.

Four or six needles united by wire to the conductor may be inserted into the aneurism; a less energetic but more disseminated effect may be thus produced.

---

* In constipation and certain irregularities of the functions of the bowels, the galvanic current is well worthy of the trouble of a trial.

After the operation, the compression below is relieved, the superior is maintained, at a degree which can be borne for a prolonged period.

If the tumor has acquired consistency, direct pressure is employed, aided by cold. The interval between the trials may be eight or ten days.

Erectile tumors should be transfixed in divers directions with long needles, which are successively put in connection with the poles of the battery.

The galvano-caustic can be placed conveniently cold upon the part to be acted upon, and cauterization effected safely and rapidly. In this way we can destroy tumors and ulcerations of the neck of the uterus, of the interior of the rectum, and of other cavities accessible to the instrument.

Such are the principal applications, at present justifiable, of electricity to medical and surgical practice.

In obstetric practice we have, I think, sufficient data to place electricity as an expedient. 1. In accidental hemorrhage. 2. In placenta prævia, when detachment of the placenta is practised. 3. In flooding post partum; and 4. In asphyxia in infants. As an emmenagogue it is one of the oldest and best recognised uses of electricity. Its application for this end may be made by the galvanic pessaries of Simpson, or in the hip-bath, in which one of the electrodes is immersed near the organs to be especially acted upon; the other handle is applied in the course of the spine, or in the bath on the opposite side of the body.

In compiling the above memoranda I have been indebted to Mattrucci's "*Lezioni di Elettricita.*" To the Instructions of the French Council of Health for the Army, published by M. Bégin in the "*Bulletin de Thérapeutique,*" and the various medical periodicals of the past two years.

Many of the applications of Electricity recommended lately, have been purposely omitted, because at present I do not think there are sufficient data to justify our recognition, and in such a matter I deem it much wiser to be somewhat barren, than *too diffuse.*

If this *brochure* should lead any *confrères* to the *cautious* and *rational* examination and employment of Electricity, Galvanism, Electro-Magnetism, or Magneto-Electricity, it will be all that I hope for.

